# The diagnostic utility of the “Thwaites’ system” and “lancet consensus scoring system” in tuberculous vs. non-tuberculous subacute and chronic meningitis: multicenter analysis of 395 adult patients

**DOI:** 10.1186/s12879-020-05502-9

**Published:** 2020-10-23

**Authors:** Tarek Sulaiman, Sai Medi, Hakan Erdem, Seniha Senbayrak, Derya Ozturk-Engin, Asuman Inan, Rok Civljak, Mihai Nechifor, Ayhan Akbulut, Alexandru Crisan, Muge Ozguler, Mustafa Namiduru, Branislava Savic, Olga Dulovic, Filiz Pehlivanoglu, Gonul Sengoz, Kadriye Yasar, Ayse Seza Inal, Emine Parlak, Isik Somuncu Johansen, Ebru Kursun, Mehmet Parlak, Emel Yilmaz, Gulden Yilmaz, Hanefi Cem Gul, Oral Oncul, Soline Siméon, Pierre Tattevin, Aysegul Ulu-Kilic, Selma Alabay, Bojana Beovic, Melanie Catroux, Yves Hansmann, Arjan Harxhi, Alper Sener, Hacer Deniz Ozkaya, Yasemin Cağ, Canan Agalar, Haluk Vahaboglu, Berna Kaya Ugur, Rodrigo Hasbun

**Affiliations:** 1 Department of Internal Medicine, Section of Infectious Diseases, UT Health McGovern Medical School, University of Texas Health Sciences Center, 6431 Fannin St. 2.112 MSB, Houston, Texas 77030m USA; 2 Department of Infectious Diseases and Clinical Microbiology, Umut Hospital, Ordu, Turkey; 3 Department of Clinical Microbiology and Infectiıus Diseases, University of Health Sciences, HaydarpaşaTraining and Research Hospital, Istanbul, Turkey; 4 Department of Clinical Microbiology and Infectiıus Diseases, University of Health Sciences, Fatih Sultan Mehmet Training and Research Hospital, Istanbul, Turkey; 5 Department of Infectious Diseases, Dr. Fran Mihaljevic University Hospital for Infectious Diseases, University of Zagreb School of Medicine, Zagreb, Croatia; 6 Department of Pharmacology, Gr. T. Popa University of Medicine and Pharmacy, Iasi, Romania; 7 Department of Infectious Diseases and Clinical Microbiology, Firat University School of Medicine, Elazig, Turkey; 8 Department of Infectious Diseases, Victor Babes University of Medicine and Pharmacy, Timisoara, Romania; 9 Medical Sciences University Elazığ Education and Research Hospital Infectious Diseases and Clinical Microbiology Department, Elazığ, Turkey; 10 Department of Infectious Diseases and Clinical Microbiology, Gaziantep University School of Medicine, Gaziantep, Turkey; 11 Institute of Microbiology and Immunology, National Reference Laboratory for Tuberculosis, Faculty of Medicine, University of Belgrade, Belgrade, Serbia; 12 Clinic for Infectious and Tropical Diseases, Clinical Centre of Serbia, Faculty of Medicine, University of Belgrade, Belgrade, Serbia; 13 Department of Clinical Microbiology and Infectiıus Diseases, University of Health Sciences, Bakırköy Dr. Sadi Konuk Training and Research Hospital, Istanbul, Turkey; 14 Department of Infectious Diseases and Clinical Microbiology, Cukurova University School of Medicine, Adana, Turkey; 15 Department of Infectious Diseases and Clinical Microbiology, Ataturk University School of Medicine, Erzurum, Turkey; 16 Department of Infectious Diseases Q, Odense University Hospital, Odense, Denmark; 17 Department of Infectious Diseases and Clinical Microbiology, Baskent University School of Medicine, Adana, Turkey; 18 Department of Infectious Diseases and Clinical Microbiology, Uludag University School of Medicine, Bursa, Turkey; 19 Department of Clinical Microbiology and Infectiıus Diseases, University of Health Sciences, GülhaneTraining and Research Hospital, Istanbul, Turkey; 20 Department of Clinical Microbiology and Infectiıus Diseases, University of Health Sciences, Gülhane Medical Faculty, Istanbul, Turkey; 21 Department of Infectious Diseases and Clinical Microbiology, Istanbul University School of Medicine, Istanbul, Turkey; 22 Department of Infectious and Tropical Diseases, University Hospital of Pontchaillou, Rennes, France; 23 Department of Infectious Diseases and Clinical Microbiology, Erciyes University School of Medicine, Kayseri, Turkey; 24 Department of Infectious Diseases, University Medical Centre, Ljubljana, Slovenia; 25 Department of Infectious Diseases, Poitiers University Hospital, Poitiers, France; 26 Department of Infectious Diseases, University Hospital, Strasbourg, France; 27 Service of Infectious Disease, University Hospital Center of Tirana, Tirana, Albania; 28 Department of Infectious Diseases and Clinical Microbiology, Onsekiz Mart University School of Medicine, Canakkale, Turkey; 29 Department of Infectious Diseases and Clinical Microbiology, Cigli Regional Education Hospital, Izmir, Turkey; 30 Department of Infectious Diseases and Clinical Microbiology, Goztepe Training and Research Hospital, Istanbul Medeniyet University, Istanbul, Turkey; 31 Department of Anesthesiology and Reanimation, Gaziantep University School of Medicine, Gaziantep, Turkey

**Keywords:** Tuberculous, Subacute, Meningitis, Thwaites, Lancet, Criteria

## Abstract

**Background:**

Tuberculous meningitis (TBM) represents a diagnostic and management challenge to clinicians. The **“**Thwaites’ system” and “Lancet consensus scoring system” are utilized to differentiate TBM from bacterial meningitis but their utility in subacute and chronic meningitis where TBM is an important consideration is unknown.

**Methods:**

A multicenter retrospective study of adults with subacute and chronic meningitis, defined by symptoms greater than 5 days and less than 30 days for subacute meningitis (SAM) and greater than 30 days for chronic meningitis (CM). The “Thwaites’ system” and “Lancet consensus scoring system” scores and the diagnostic accuracy by sensitivity, specificity, and area under the curve of receiver operating curve (AUC-ROC) were calculated. The “Thwaites’ system” and “Lancet consensus scoring system” suggest a high probability of TBM with scores ≤4, and with scores of ≥12, respectively.

**Results:**

A total of 395 patients were identified; 313 (79.2%) had subacute and 82 (20.8%) with chronic meningitis. Patients with chronic meningitis were more likely caused by tuberculosis and had higher rates of HIV infection (*P* < 0.001). A total of 162 patients with TBM and 233 patients with non-TBM had unknown (140, 60.1%), fungal (41, 17.6%), viral (29, 12.4%), miscellaneous (16, 6.7%), and bacterial (7, 3.0%) etiologies. TMB patients were older and presented with lower Glasgow coma scores, lower CSF glucose and higher CSF protein (*P* < 0.001). Both criteria were able to distinguish TBM from bacterial meningitis; only the Lancet score was able to differentiate TBM from fungal, viral, and unknown etiologies even though significant overlap occurred between the etiologies (*P* < .001). Both criteria showed poor diagnostic accuracy to distinguish TBM from non-TBM etiologies (AUC-ROC was <. 5), but Lancet consensus scoring system was fair in diagnosing TBM (AUC-ROC was .738), sensitivity of 50%, and specificity of 89.3%.

**Conclusion:**

Both criteria can be helpful in distinguishing TBM from bacterial meningitis, but only the Lancet consensus scoring system can help differentiate TBM from meningitis caused by fungal, viral and unknown etiologies even though significant overlap occurs and the overall diagnostic accuracy of both criteria were either poor or fair.

## Background

Meningitis can be categorized as acute and subacute based on duration of symptoms [1]. Subacute meningitis (SAM) is commonly defined as inflammation evolving for greater than 5 days and less than 30 days and chronic meningitis (CM) as greater than 30 days without resolution of cerebrospinal fluid (CSF) abnormalities [1]. The majority of adult patients with community-acquired meningitis (CAM) is admitted and receives empiric antimicrobial therapy pending the results of CSF cultures [2]. Once the CSF bacterial cultures are negative, the decision to empirically start anti-mycobacterial therapy for suspected tuberculous meningitis (TBM) is difficult as laboratory tests such as the CSF acid fast bacilli (AFB) smears and cultures are very insensitive and delays in therapy are associated with death [3–5]. TBM is reported in up to 1% of all tuberculosis cases [6] and is second most common cause of community-acquired meningitis in a recent international study [7]. TBM usually presents with a subacute presentation with variable neurologic manifestations, including meningitis, meningoencephalitis, cranial nerve involvement, myelitis, radiculopathy, neuropathy, depression, paraplegia, stroke, and abscess formation [8, 9]. The low sensitivity and delays of the current microbiological techniques makes TBM a diagnostic and management challenge that fostered the development of the “Thwaites’ system” and “Lancet consensus scoring system” [4, 5].

The study objectives: a) to explore the sensitivity and specificity of the two commonly used methods -Thwaites’ scoring system (Table 1) [4], and more recently, the Lancet consensus scoring system (Table 2) [5] in diagnosing TBM. b) To explore if both scoring systems were able to differentiate TBM from other etiologies of SAM & CM. The need and the purpose of this study is to help clinicians to determine if TBM should be suspected and empirically treated. Two commonly used methods -Thwaites’ scoring system (Table 1) [4], and more recently, the Lancet consensus scoring system (Table 2) have been developed to help determine the probability of TBM and to help clinicians determine if TBM should be suspected and empirically treated [5]. The scoring systems include clinical features, CSF findings, as well as neurological imaging in making a diagnosis. This study was designed to explore the diagnostic utility of the “Thwaites’ system” and “Lancet consensus scoring system” in differentiating TBM from the other more common etiologies of SAM where TBM is an important consideration.

**Table 1:** Thwaites scoring system

	Score
**Age (years)**	
≥ 36	2
< 36	0
**WBC (10**^**3**^ **/ml)**	
≥ 15,000	**4**
< 15,000	**0**
**History of illness (days)**	
≥ 6	**5**
< 6	**0**
**CSF total WBC (10**^**3**^ **/ml)**	
≥ 900	3
< 900	0
**CSF % neutrophils**	
≥ 75	4
< 75	0

*WBC* White blood cell count, *CSF* Cerebrospinal fluid

**Table 2:** Lancet scoring system

**Clinical criteria**	**Score (Maximum category score = 6)**
Symptom duration of more than 5 days	4
Systemic symptoms suggestive of tuberculosis (one or more of the following): weight loss (or poor weight gain in children), night sweats, or persistent cough for more than 2 weeks	2
History of recent (within past year) close contact with an individual with pulmonary tuberculosis or a positive TST or IGRA (only in children < 10 years of age)	2
Focal neurological deficit (excluding cranial nerve palsies)	1
Cranial nerve palsy	1
Altered consciousness	1
**CSF criteria**	**(Maximum category score = 4)**
Clear appearance	1
Cells: 10–500 per μl	1
Lymphocytic predominance (> 50%)	1
Protein concentration greater than 1 g/L	1
CSF to plasma glucose ratio of less than 50% or an absolute CSF glucose concentration less than 2·2 mmol/L	1
**Cerebral imaging criteria**	**(Maximum category score = 6)**
Hydrocephalus	1
Basal meningeal enhancement	2
Tuberculoma	2
Infarct	1
Pre-contrast basal hyperdensity	2
**Evidence of tuberculosis elsewhere**	**(Maximum category score = 4)**
Chest radiograph suggestive of active tuberculosis: signs of tuberculosis = 2; miliary tuberculosis = 4	2/4
CT/ MRI/ ultrasound evidence for tuberculosis outside the CNS	2
AFB identified or *Mycobacterium tuberculosis* cultured from another source—i.e., sputum lymph node, gastric washing, urine, blood culture	4
Positive commercial *M tuberculosis* NAAT from extra-neural specimen	4

Exclusion of alternative diagnoses

An alternative diagnosis must be confirmed microbiologically (by stain, culture, or NAAT when appropriate), serologically (eg, syphilis), or histopathologically (eg, lymphoma). The list of alternative diagnoses that should be considered, dependent upon age, immune status, and geographical region, include: pyogenic bacterial meningitis, cryptococcal meningitis, syphilitic meningitis, viral meningo-encephalitis, cerebral malaria, parasitic or eosinophilic meningitis (*Angiostrongylus cantonesis, Gnathostoma spinigerum*, toxocariasis, cysticercosis), cerebral toxoplasmosis and bacterial brain abscess (space-occupying lesion on cerebral imaging) and malignancy (eg, lymphoma)

*TST* tuberculin skin test, *IGRA* interferon-gamma release assay, *NAAT* nucleic acid amplification test, *AFB* acid-fast bacilli. The individual points for each criterion (one, two, or four points) were determined by consensus and by considering their quantified diagnostic value as defined in studies

## Methods

### Case definition and data collection

**Fig. 1:**
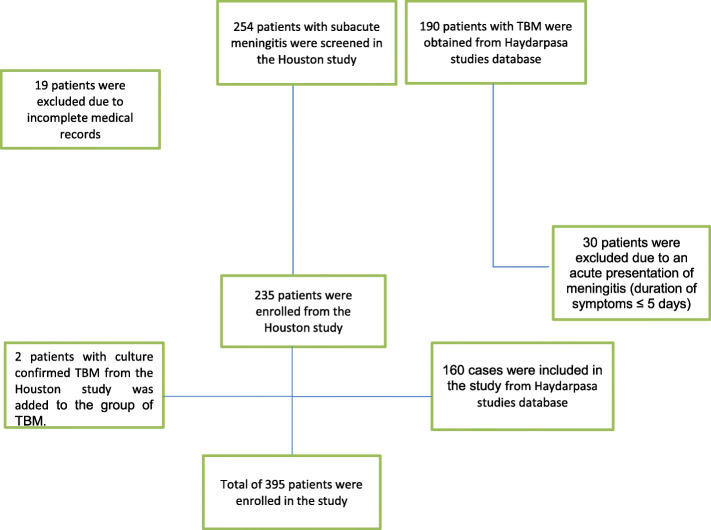
Cohort assembly

We conducted a multicenter retrospective study of 395 adults with subacute and chronic meningitis (see Fig. 1). SAM is commonly defined as inflammation evolving for greater than 5 days and less than 30 days and CM as greater than 30 days. Data was collected through ICD then chart review and microbiology data extraction.

Inclusion Criteria: a) adult patient (age > 14 years); b) symptoms of meningitis (fever, headache, stiff neck, altered mental status or focal neurological symptoms); c) duration of symptoms more than 5 days; d) CSF white cell count > 5 cells/mm^3^ [1]. A total of 233 non TBM-patients were identified who presented to an emergency department (ED) between January 1, 2005 and January 1, 2015 at 9 Memorial Hermann hospitals and to Lyndon B Johnson Hospital in Houston, Texas. A total of 162 TBM microbiologically confirmed patients were identified by at least one of the following tests on the CSF was mandatory for microbiological confirmation; a positive Ehrlich-Ziehl-Neelsen stain, positive *Mycobacterium tuberculosis* (Mtb) culture, or positive Mtb-PCR.160 TBM from Haydarpasa studies database that involved patients with TBM in Turkey (*n* = 98), Croatia (*n* = 19), Romania (*n* = 18), Serbia (*n* = 16), Denmark (*n* = 5), Slovenia (*n* = 2), France (*n* = 1), and Albania (*n* = 1) between 2000 and 2012 [3] and 2 patients from our study in Houston.

The study was approved by the University of Texas Health in Houston Committee for the Protection of Human Subjects, by the Memorial Hermann Hospital Research Review Committee and by the Harris Health Research and Sponsored Programs department and by the review committees of all the Haydarpasa study centers.

### Etiologies, “Thwaites’ system” and “Lancet consensus scoring system”

Etiologies of the patients were divided into six categories: a) tuberculosis; b) fungal; c) viral; d) bacterial; e) miscellaneous; f) unknown (Table 3). For TBM patient’s duration of symptoms ranged between 6 to 356 days, the acid-fast bacilli was seen in the CSF samples of 33 patients (8 patients tested positive by culture). *Mycobacterium tuberculosis* was cultured from the CSF samples of 117 patients (14 patients tested positive by PCR). The CSF samples of 33 patients tested positive by PCR (3 were acid-fast bacillus smear positive and 29 acid-fast bacillus smear negative). A total of 106 isolates were tested by the solid culture proportion method on Löwenstein-Jensen medium (*n* = 102) and on Middlebrook 7H10 agar (*n* = 4) using the standard protocol. The 49 isolates were tested using automated culture systems including BACTEC MGIT 960 (*n* = 46) and BACTEC 9000 MB (*n* = 3). The acid-fast bacilli was seen in other sterile body fluids and tissue samples of 8 patients. *Mycobacterium tuberculosis* was cultured also from other sterile body fluids and tissue samples of 16 patients. Acid-fast bacilli was seen in histopathological examination of 7 patients.

**Table 3:** A comparison of the baseline characteristics between subacute and chronic meningitis and between tuberculous and non-tuberculous meningitis

Clinical Features	Subacute Meningitis^**a**^ (***n*** = 313)	Chronic Meningitis^**b**^ (***n*** = 82)	***P*** value	Tuberculous meningitis (***n*** = 160)	Non-Tuberculous Meningitis (***n*** = 235)	***P***- value^**c**^
**Median age in years (range)**	**38 (14–82)**	**41 (15–76)**	**0.104**	**36 (14–82)**	**40 (18–78)**	**< 0.001**
**HIV/AIDS** ^**d**^**, n (%)**	**108/313 (34.5)**	**43/82 (52.4)**	**0.003**	**110/160 (68.8)**	**41/235 (17.4)**	**< 0.001**
**Presenting Symptoms, n (%)**						
**Duration of symptoms (days), range**	**10 (2–28)**	**41 (30–356)**	**< 0.001**	**15 (6–356)**	**8 (2–30)**	**< 0.001**
**Fever**	**209/313 (66.7)**	**55/82 (67.1)**	**0.959**	**114/160 (71.3)**	**150/235 (63.8)**	**0.124**
**Headache**	**262/311(84.2)**	**54/82 (65.9)**	**< 0.001**	**129/158 (81.6)**	**187/235 (79.6)**	**0.612**
**Nausea/vomiting**	**180/311 (57.9)**	**44/82 (53.6)**	**0.492**	**48/102 (47.1)**	**45/89 (50.6)**	**0.63**
**Median GCS** ^**e**^ **(range)**	**15 (3–15)**	**15 (3–15)**	**0.100**	**11 (3–15)**	**15 (3–15)**	**< 0.001**
**CSF Profile**						
**CSF WBC** ^**f**^	**150 (3–3405)**	**120 (2570)**	**0.526**	**161 (2–2570)**	**84 (5_3405)**	**0.364**
**CSF protein, mg/dl**	**108 (21–3500)**	**131 (21–1900)**	**0.381**	**188 (21–3500)**	**87 (22–466)**	**< 0.001**
**CSF glucose, mg/dl**	**46 (14–193)**	**27 (0–81)**	**< 0.001**	**27 (0–115)**	**52 (1–193)**	**< 0.001**
**Tuberculous meningitis**	**104/313 (33.2)**	**56/82 (68.3)**	**< 0.001**	**N/A**	**N/A**	**N/A**

^a^Subacute meningitis defined as duration of symptoms between 5 and 29 days

^b^chronic meningitis defined as duration of symptoms > 30 days

^c^All statistically significant outcomes signified by bolding the *P* value

^d^Human immunodeficiency virus/Acquired Immunodeficiency Syndrome

^e^Glasgow Coma scale

^f^White blood cell counts

Fungal meningitis was identified by positive CSF antigens and/or fungal CSF cultures. Viral meningitis was identified by molecular methods: positive polymerase chain reaction (PCR) in CSF or by positive arboviral serologies. Bacterial meningitis was documented by positive CSF cultures. Miscellaneous etiologies of meningitis (noninfectious and parasites) and were identified by positive histopathology in brain biopsy results or positive serologies.

We scored all patients with SAM and CM using the “Thwaites’ system” and “Lancet consensus scoring system,” and compared the scores of TBM patients with the non-TBM. The Thwaites’ system has 5 parameters including age, duration of illness, total white blood cell count, CSF cell count and the CSF neutrophilic percent, with a maximum score of 13. The patient is classified as possible TBM with a total score of 4 or less, and with possible bacterial meningitis if the score is greater than 4 (Table 1) [4].

The Lancet consensus scoring system has 20 parameters, which are divided in 4 categories (clinical, CSF, CNS imaging and evidence of TB elsewhere) with a maximum score of 20 [5]. A definite diagnosis of TBM is made if there is evidence of AFB in CSF smear, culture or on histopathology of brain or spinal cord. A probable diagnosis is made if the total score is > 10 pts. if patients with no imaging, or > 12 pts. with imaging. A possible diagnosis is made with scores between 6 and 9 without imaging or 6–11 with imaging. Based on the total scores assigned, the diagnosis of TBM is either definite, probable, possible or no TBM (Table 2) [5].

### Statistical analysis

An analysis of variance analysis was used to compare the median values of the “Thwaites’ system” and “Lancet consensus scoring system” between TBM and the other etiological groups with a *P* value < 0.05 being considered significant. Areas under the curve – Receiver Operating Curve (AUC-ROC) of both scores for all etiologies of SAM were calculated. All analysis was performed using SPSS version 25 (IBM, Austin, TX, USA).

## Results

### Study population

We screened 254 patients with SAM in the Houston study; after excluding 19 patients due to incomplete medical records a total of 235 patients were enrolled (see Fig. 1). Of those 233 patients had non-TBM. A total of 190 patients with TBM were obtained from Haydarpasa studies database [3], after excluding 30 patients due to an acute presentation (duration of symptoms ≤5 days) a total of 160 cases were included in the study. Furthermore, two patients with culture confirmed TBM from the Houston study was added to the group of TBM. A total of 395 patients were identified; 313 (79.2%) had subacute and 82 (20.8%) with chronic meningitis (see Table 3). Patients with chronic meningitis were more likely caused by tuberculosis and had higher rates of HIV infection (*P* < 0.001), while TMB patients were older and presented with lower Glasgow coma scores, lower CSF glucose and higher CSF protein (*P* < 0.001).

### Etiologies

A total of 162 (41.0%) patients had TBM and 233 (59.0%) patients had non-TBM. Fungal meningitis was diagnosed in 17.6% (41/233) and included: 36 cases of *Cryptococcus neoformans*; 3 cases of *Coccidiodes immitis*; and two cases of *Histoplasma capsulatum* meningitis. Viral meningitis was observed in 12.4% (29/233) and included: 10 cases of Herpes simplex virus (HSV) 1&2; 8 cases of West Nile virus; 4 cases of *Varicella*-Zoster virus (VZV); 3 cases of Saint Louis virus; 2 cases of Enterovirus; and 2 cases of Human Immunodeficiency Virus (HIV) infection. Bacterial meningitis was diagnosed in 3.0% (7/233) and included: 3 cases of *Streptococcus pneumoniae*; 1 case of methicillin susceptible *Staphylococcus aureus*; 1 case of *Haemophilus influenzae;* 1 case of *Streptococcus pyogenes;* and 1 case of coagulase negative *staphylococcus*. A total of 6.9% (16/233) of patients had miscellaneous etiologies of meningitis (noninfectious and parasites) and included: 5 cases of systemic lupus erythematosus (SLE) meningoencephalitis, 2 cases of paraneoplastic syndromes: 1 case of Breast cancer (positive anti Yo antibodies, CSF lymphocytic pleocytosis, and negative CSF cultures) and 1 case of anti NMDA (N-methyl D-aspartate); 2 cases of neurosarcoidosis; 1 case of meningeal carcinomatosis; 1 case of acute disseminated encephalomyelitis (ADEM); 1 case of central nervous system lymphoma and other infectious parasitic etiologies: 2 cases of cerebral toxoplasmosis and 2 cases of neurocysticercosis. An unknown etiology was seen in 60.1% (140/233). (Table 4).

**Table 4:** Etiologies, “Thwaites system and Lancet consensus scoring system” in 395 adults with subacute/chronic meningitis

Etiology	Thwaites Scoring Classification^a^		Lancet Scoring Classification^b^			
(*n* = 395)	≤4 points (Possible TBM)	> 4 points (Possible BM)	Definite TBM Positive TB Culture	Probable ≥12 points	Possible 6─11 points	No TBM < 6 points
TBM^c^ (162)	160	2	162	81	81	0
Fungal^d^ (41)	41	0	0	5	35	1
Viral^e^ (29)	29	0	0	0	28	1
Bacterial^f^ (7)	6	1	0	0	7	0
Miscellaneous^g^ (16)	16	0	0	6	10	0
Unknown^h^ (140)	139	1	0	14	123	3

^a^If a patient has a total score of 4 or less, the patient is classified as tubercular meningitis (TBM) and a score of more than 4 is suggestive of bacterial meningitis

^b^Definite diagnosis of TBM is made if there is evidence of Acid Fast Bacilli (AFB) in CSF smear, culture or on histopathology of brain or spinal cord. A probable diagnosis is made if the total score is >10 points if patients have no imaging, or >11 points if imaging was used. A possible diagnosis is made with scores between 6-9 points without imaging or 6-11 with imaging. No TBM if the total score < 6 points

^c^162 cases of CSF Culture positive for TB Complex, although all 162 cases are definite TBM , we calculated the actual Lancet score

^d^36 cases of Cryptococcal Meningitis , 3 cases Coccidiosis Meningitis, 2 cases of Histoplasma Meningitis

^e^10 cases of Herpes simplex 1 & 2, 8 cases of West Nile Virus, 4 cases of Varicella Zoster Virus, 3 cases of Saint Louis Virus, 2 Cases of Enterovirus, 2 cases of acute HIV

^f^3 cases of *Streptococcus pneumoniae* , 1 case of *Methicillin Sensitive Staphylococcus Aureus* , 1 case of *Haemophilus influenzae* , 1 case of Group A Streptococcus , 1 case of coagulase negative staphylococcus

^g^Miscellaneous etiologies includes: non-infectious etiologies: 5 cases of Systemic lupus erythematosus meningoencephalitis, 2 cases of para neoplastic (1 case due Breast cancer, 1 case due to anti NMDA), 2 cases of Neurosarcoidosis, 1 case of Meningeal Carcinomatosis, 1 case of disseminated encephalomyelitis (ADEM), 1 case of Central Nervous System Lymphoma. Parasitic infections etiologies: 2 cases of Cerebral Toxoplasmosis, 2 cases of Neurocysticercosis

^h^Unknown cause of meningitis

### “Thwaites’ system” and “Lancet consensus scoring system” in subacute meningitis

All enrolled patients (*n* = 395) were scored with the “Thwaites’ system” and “Lancet consensus scoring system” (see Table 5). The majority of patients (*n* = 391,99%) scored ≤4 in Thwaites scoring system, only four patients scored > 4, two TBM cases, a bacterial case and one unknown etiology. Regarding Lancet scoring system, TBM cases consisted of 162 patients: 81 cases were classified as possible, and 81 cases as probable TBM. Fungal cases consisted of 41 patients: 35 cases were classified as possible, five cases as probable, and one case as no TBM. Viral cases consisted of 29 patients: 28 cases were classified as possible and one case classified as no TBM. Bacterial cases consisted of seven patients which all were classified as possible – one of which also scored Thwaites > 4. Miscellaneous cases (noninfectious and parasitic) consisted of 16 patients: six cases were classified as probable and ten cases as possible. Unknown etiology cases consisted of 140 patients: 14 cases were classified as probable, 123 cases as possible, and three cases as no TBM including a case scoring > 4 with Thwaites as well.

**Table 5:** A and B: Sensitivity, specificity, and predictive values of Thwaites scoring systems ≤4 and the Lancet scoring system ≥12 between patients with tuberculous meningitis and other etiologies

**A. Thwaites scoring system**				
**Etiology**	**Sensitivity (%)**	**Specificity (%)**	**Positive Predictive Value (%)**	**Negative Predictive Value (%)**
**A. Thwaites**				
**Tuberculosis**	1.2	99.1	50	59.1
**Fungal**	0	98.9	0	89.5
**Viral**	0	98.9	0	92.6
**Bacterial**	14.3	99.2	25	98.5
**Miscellaneous**	0	98.9	0	95.9
**Unknown**	0.7	98.8	25	65.2
**B. Lancet Score**				
**Tuberculosis**	50	89.3	76.1	72
**Fungal**	12.2	71.5	4.7	87.5
**Viral**	0	71.1	0	90
**Bacterial**	0	97.9	0	97.6
**Miscellaneous**	37.5	73.6	5.7	96.5
**Unknown**	10	63.9	13.2	56.4

**Fig. 2:**
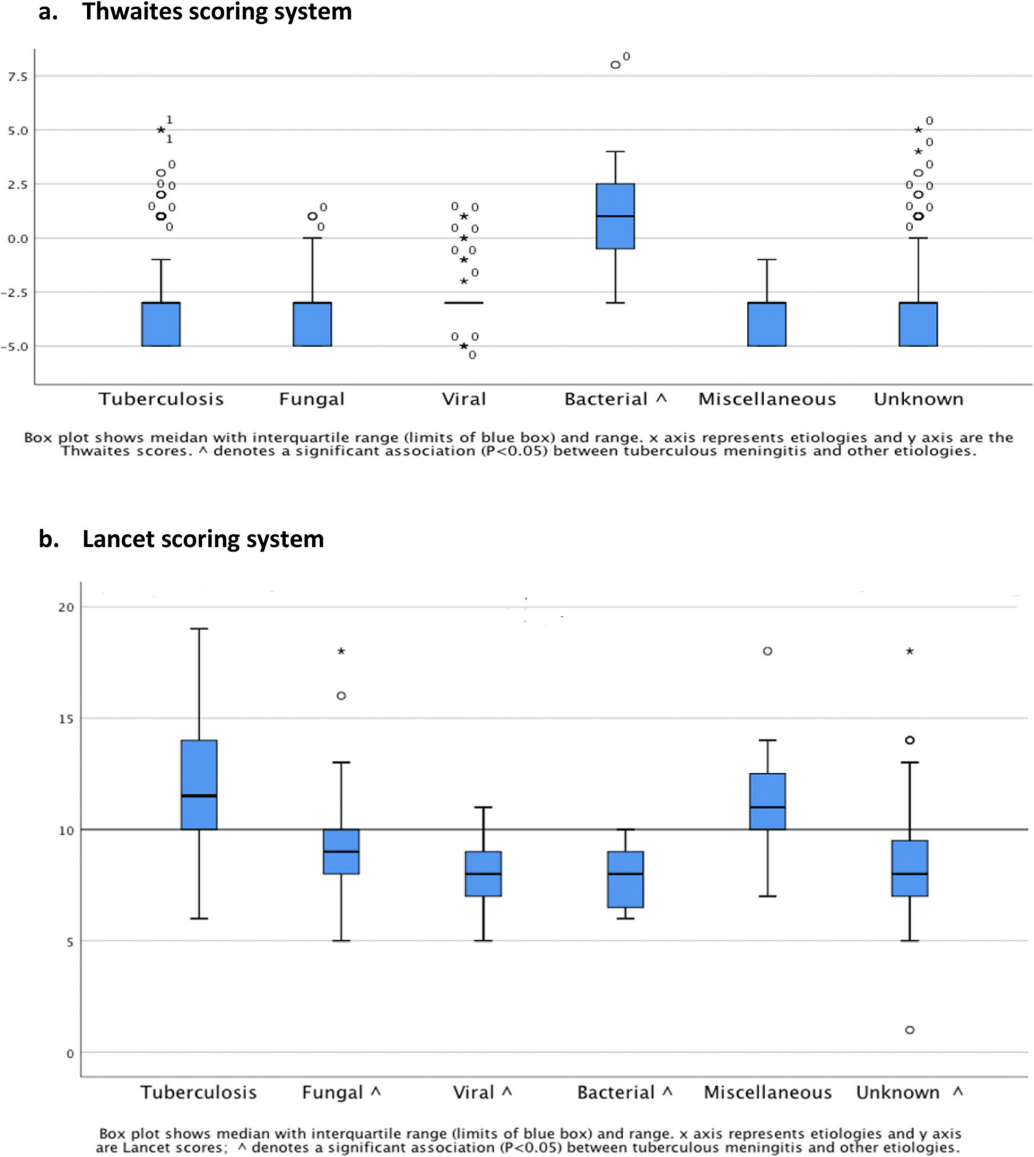
Comparison of the “Thwaites‘system” and “Lancet consensus scoring system” between patients with tuberculous meningitis and other etiologies

TBM cases (*n* = 162) scored with Thwaites system, showed a median of − 3 (− 5.0 ─ 5.0) and Lancet scoring system, showed a median 12 (6.0–19.0) (see Table 5). The Thwaites scoring system was able to distinguish TBM from bacterial meningitis [median 1.0 (− 3.0–8.0), (*P* < .001)], but it was not able to distinguish TBM from viral meningitis [median − 3 (− 5.0–1.0), (*P* = .281)], fungal meningitis [median − 3 (− 5.0–1.0) (*P* = .284)], unknown causes of meningitis [median − 3 (− 5.0–5.0), (*P* = .939)], and miscellaneous causes of SAM and CM [median − 3 (− 5 − − 1), (*P* = .287)]. (Fig. 2a)**.** The Lancet scoring system was able to distinguish TBM from viral meningitis (*P* < .001) median 8 (5–11), fungal meningitis (*P* < .001) median 9 (5–18), bacterial meningitis (*P* < .001) median 8 (6–10), unknown causes of meningitis (*P* < .001), median 8 (1–18), and was not able to distinguish TBM form miscellaneous causes of meningitis (*P* = .255) median 11 (7–18), (Fig. 2b).

**Fig. 3:**
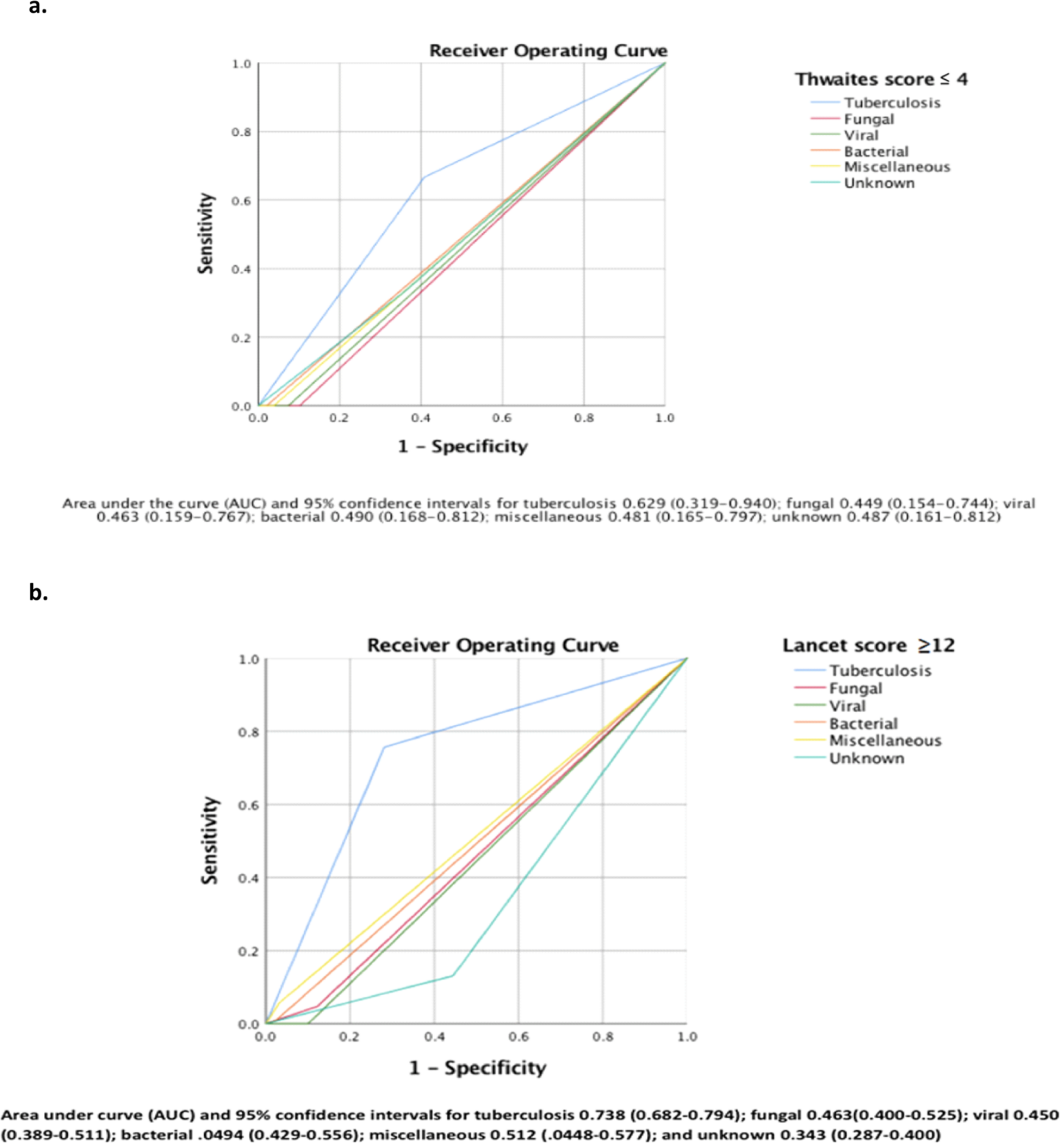
Diagnostic accuracy of a Thwaites score ≤ 4 and Lancet score ≥ 12 in tuberculous meningitis and other etiologies. **a**. Thwaites scoring system. **b**. Lancet scoring system

Our results showed that the diagnostic accuracy for the Thwaites scoring system in diagnosing TBM was poor, and it was unable to distinguish TBM from non- TBM etiologies: fungal, viral, bacterial, miscellaneous, and unknown etiologies (Fig. 3a). The diagnostic accuracy of the Lancet scoring system in diagnosing TBM was fair, but it was unable to distinguish TBM from non-TBM etiologies: fungal, viral, bacterial, miscellaneous, and from unknown etiologies (Fig. 3b).

## Discussion

This study was conducted to evaluate the diagnostic utility of the “Thwaites’ system” and “Lancet consensus scoring system” in SAM and CM caused by different etiologies other than tuberculosis. TBM is reported in up to 1% of all tuberculosis cases [6] and is second most common cause of community-acquired meningitis in a recent international study [7]. TBM usually presents with a subacute or chronic presentation with variable neurologic manifestations, including meningitis, meningoencephalitis, cranial nerve involvement, myelitis, radiculopathy, neuropathy, depression, paraplegia, stroke, and abscess formation [8, 9]. In this study, TBM presented more commonly with chronic meningitis with higher rates of HIV coinfection. The low sensitivity and delays of the current microbiological techniques makes TBM a diagnostic and management challenge that fostered the development of the “Thwaites’ system” and “Lancet consensus scoring system” [4, 5]. Studies showed that detection of microorganisms in CSF samples by microscopy or culture techniques is crucial for the differential diagnosis of TBM and bacterial meningitis [10, 11]. Thwaites established a scoring system employing 143 cases of TBM and 108 cases of bacterial meningitis by regression analysis; this scoring system was evaluated by Sunbul et al. [12] using 23 cases of TBM and 103 cases of bacterial meningitis. Their evaluation revealed the sensitivity and specificity of the system to be 95.6 and 70.8%, respectively. Zhang et al. evaluated Thwaites scoring system and concluded to be highly effective for the differential diagnosis of TBM and initially treated bacterial meningitis but were found to be less effective for that of TBM and partially treated bacterial meningitis [13]. J. S Sebastian et al. evaluated Thwaites scoring system in 527 patients (adults and pediatrics), and concluded that the scoring system was sensitive but not specific when used to distinguish TBM from bacterial meningitis in HIV negative adults. In HIV positive adults the index had low diagnostic accuracy [13].

In our study, the Thwaites scoring system scored < 4 (391, 99%), with only 4 patients scoring higher than 4, two patients with TBM, one with pneumococcal meningitis case, and the other with unknown etiology. Our results show that the Thwaites scoring system was poor to distinguish TBM from other etiologies of SAM and CM (AUC-ROC <.5) (Fig. 3a). Unlike the mentioned studies [12–14], our findings of poor sensitivity (1.2%) and high specificity (99.1%) were noticed for Thwaites system in diagnosing TBM (Table 4) because all enrolled patients presented with symptoms more than 5 days, which scores − 5 from the total score, in addition the majority of cases are not due to bacterial meningitis and few had a serum leukocyte count > 15,000 (cells /μL) and/or a CSF Leukocyte count > 900 (cells/μL) (data not shown). Despite the poor diagnostic accuracy, the Thwaites system was able to distinguish subacute bacterial meningitis from TBM (*P* < .001), but not to distinguish TBM from viral, fungal, unknown and miscellaneous causes of SAM.

Both the “Thwaites’ system” and “Lancet consensus scoring system” were evaluated by Erdem [15] to distinguish TBM from Brucella meningoencephalitis (BME), which is also complex to diagnose and found that Thwaites scoring system more frequently predicted BME cases (*n* = 292, 99.3%) compared to the TBM group (*n* = 182, 95.8%) (*P* = .017). According to the Lancet scoring system, the mean scores for BME and TBM were 9.43 _ + 1.71 and 11.45 _ + 3.01, respectively (*P* < .001). In addition, TBM cases were classified into “probable” category more significantly compared to BME cases, and BME cases were categorized into the “possible” category more frequently [15]. In our study, the Lancet scoring system was able to differentiate TBM from fungal meningitis (*P* < .001), viral meningitis (*P* < .001), subacute bacterial meningitis (*P* < .001), unknown causes of subacute meningitis (*P* < .001), but was not able to differentiate TBM from miscellaneous causes of subacute meningitis (*P* = .255).

Out of 162 patients with TBM, 81 cases were classified as possible (< 12 points), and 81 cases were classified as probable (≥12 points). Based on the cut off (≥12 points), the diagnostic accuracy was fair in diagnosing TBM, (AUC-ROC = .738), this finding could be due to 50% of the patients with TBM scored “possible” based on the current cutoff. The ability of the score to distinguish TBM from other etiologies of SAM and CM was poor (AUC-ROC <.5); this could be due to the cutoff value of 12 points with significant overlap (≥12 points) of the patients with non-TBM etiologies. This cut off score was exceeded frequently by fungal and miscellaneous and unknown etiologies. Miscellaneous causes of SAM group consisted of 16 patients, 10 were classified into “possible” category, and 6 patients into “probable” category (2 cases of neurocysticercosis, 2 cases of Toxoplasmosis, 1 case of meningeal carcinomatosis, 1 case of neurosarcoidosis), although Lancet scoring system was able to differentiate TBM from fungal meningitis but overlap were noticed, 5 patients (12.2%) were classified into “probable” (2 cases of Cryptococcus, 2 cases of Coccidioides, 1 case of Histoplasma). Of the unknown causes of subacute meningitis group, 14 patients (10.0%) were classified into “probable” group. None of the viral or bacterial cases were classified as “probable”.

Although the Lancet scoring system was able to distinguish TBM (except from the miscellaneous causes), we advise to keep in mind other etiologies in addition to TBM when the microbiological diagnosis of SAM and CM is not achieved, especially noninfectious etiologies and fungal meningitis. Anti-NMDA meningoencephalitis was first described in 2005 as a syndrome of psychiatric symptoms and neurologic sequelae associated with ovarian teratomas [16], in our study only 7 patients were tested making this an under diagnosed etiology.

Our study had limitations. First, the majority of the patients in our study had unknown etiology (140, 60.1%), due to the retrospective design of the study the diagnostic testing was not comprehensive. This is the unfortunate reality in community-acquired meningitis as other studies have shown [2, 17–19]. Secondly, the small subgroup of etiologies such as bacterial meningitis may affect the power to detect a difference. Thirdly, limitation in using Thwaites system as there are − 5 points for those with ≥6 days since symptom presentation (all enrolled patients). Fourthly, two very distinct populations (US and eastern European/middle east) were used to gather samples and the latter where the majority of TBM cases emerged.

Despite these limitations, our study had several strengths. This study represents the first evaluation of the utility of the “Thwaites’ system” and “Lancet consensus scoring system” in SAM and CM and highlights the importance to take into account other etiologies especially in the setting of possible TBM by the Lancet consensus scoring system.

## Conclusion

The “Thwaites’ system” and “Lancet consensus scoring system” showed poor diagnostic accuracy to distinguish TBM from other causes of SAM and CM. Other etiologies should be considered especially in patients with possible TBM by Lancet criteria. Novel CSF molecular diagnostic methods may increase the yield to identify the etiologies and ultimately improve care.

## Data Availability

The datasets used and/or analyzed during the current study. are available from the corresponding author on a reasonable request.

## Abbreviations

TBM: Tuberculous meningitis
SAM: Subacute meningitis
CM: Chronic meningitis
AUC-ROC: Area under the curve of receiver operating curve
CSF: Cerebrospinal fluid
CAM: Community-acquired meningitis
AFB: Acid fast bacilli
VZV: *Varicella*-Zoster virus
HIV: Human Immunodeficiency Virus infection
SLE: Systemic lupus erythematosus
N-methyl D-aspartate: Anti NMDA
ADEM: Acute disseminated encephalomyelitis
